# Long descending commissural V0v neurons ensure coordinated swimming movements along the body axis in larval zebrafish

**DOI:** 10.1038/s41598-022-08283-0

**Published:** 2022-03-14

**Authors:** Kohei Kawano, Kagayaki Kato, Takumi Sugioka, Yukiko Kimura, Masashi Tanimoto, Shin-ichi Higashijima

**Affiliations:** 1 National Institutes of Natural Sciences, Exploratory Research Center On Life and Living Systems (ExCELLS), National Institute for Basic Biology, Higashiyama 5-1, Myodaiji, Okazaki, Aichi 444-8787 Japan; 2 Graduate University for Advanced Studies (SOKENDAI), Okazaki, Aichi 444-8787 Japan

**Keywords:** Neuroscience, Zoology

## Abstract

Developmental maturation occurs in slow swimming behavior in larval zebrafish; older larvae acquire the ability to perform slow swimming while keeping their head stable in the yaw dimension. A class of long-distance descending commissural excitatory V0v neurons, called MCoD neurons, are known to develop in a later phase of neurogenesis, and participate in slow swimming in older larvae. We hypothesized that these MCoD neurons play a role in coordinating the activities of trunk muscles in the diagonal dimension (e.g., the rostral left and the caudal right) to produce the S-shaped swimming form that contributes to the stability of the head. Here, we show that MCoD neurons do indeed play this role. In larvae in which MCoD neurons were laser-ablated, the swimming body form often adopted a one-sided (C-shaped) bend with reduced appearance of the normal S-shaped bend. With this change in swimming form, the MCoD-ablated larvae exhibited a greater degree of head yaw displacement during slow swimming. In mice, the long-distance descending commissural V0v neurons have been implicated in diagonal interlimb coordination during walking. Together with this, our study suggests that the long-distance descending commissural V0v neurons form an evolutionarily conserved pathway in the spinal locomotor circuits that coordinates the movements of the diagonal body/limb muscles.

## Introduction

In the early developmental stage, most animals can only exhibit immature forms of behaviors. As development progresses, they acquire the ability to produce more mature or refined forms of behaviors^1–3^. Concurrent with such changes, many new connections are formed in the nervous system^4,5^, which suggests that the formation of new connections is linked to the developmental maturation of behaviors. In animals in which new neurons are generated during development (e.g., fish and amphibians^1,6–8^), the incorporation of new neurons into the pre-existing circuits together with the forming of new connections likely contributes to the maturation of movements.

One example of this developmental maturation of behaviors is seen in the swimming behavior of larval zebrafish. Older larvae (i.e., 4–5 dpf) exhibit more refined forms of swimming than younger larvae (i.e., 2 dpf)^9^. During this period (from 2 dpf to 4–5 dpf), new neurons are generated in both the brain and spinal cord^9,10^, with the latter being mainly responsible for generating swimming outputs^11^. One class of premotor spinal neurons that are added later to the early spinal neuronal circuits are MCoD neurons, a subclass of V0v neurons (V0v neurons represent an excitatory class of neurons derived from the p0 developmental domain of the spinal cord)^12^. MCoD neurons are absent in the early stages and develop in a later phase of neurogenesis in the spinal cord^9,12^. MCoD neurons are active during slow swimming^9,13^. As for their function, one study showed that they contribute to the general excitability of spinal swimming circuits, and their ablation decreased the occurrence frequency of spontaneous swimming^14^. Their function in the more specific aspect of slow swimming remains elusive, however.

**Figure 1:**
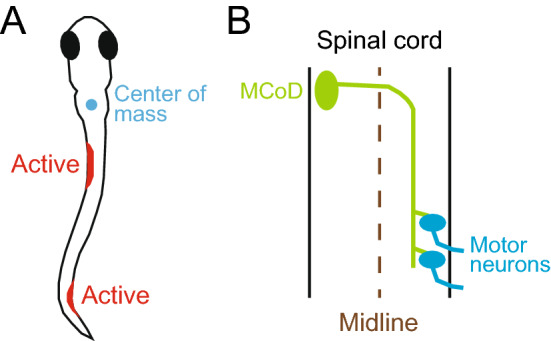
Swim form of a zebrafish larva and projection of an MCoD neuron. (**A**) One of the swim forms of a zebrafish larva at 4 to 5 dpf. The cyan circle shows the center of mass, which is located near the swim bladder. Muscle contractions are presumed to occur in the two locations marked in red. (**B**) Projection pattern of an MCoD neuron in the spinal cord. The axon of the MCoD neuron crosses the midline (broken line), descends on the contralateral spinal cord, and makes mono-synaptic excitatory connections onto caudally located MNs.

One of the most characteristic features of slow swimming in older larvae is the stability of the head in the yaw dimension^9^. In slow swimming, the muscle contractions are mostly confined to the trunk that is caudal to the swim bladder^9^. Given that the center of the mass is located near the swim bladder in larval zebrafish^15^ (Fig. 1A), it is thought that the head yaw displacement is produced by the recoil of the yawing moment force generated in the trunk. Considering this, the stability of the head yaw indicates that the net yawing moment force in the trunk that acts to the center of the mass is very small during slow swimming. Swimming consists of a descending wave of muscle contraction along the trunk. With this movement, the bending of the body transmits force to the surrounding water, and this region of the body, in turn, receives reaction force. To make the net yawing moment force minimal, the movements of the rostral and caudal parts of the trunk need to be highly coordinated with the diagonal dimension; when the rostral part receives leftward force, for example, the caudal part needs to receive rightward force. For this to occur, the swimming body form cannot be C-shaped (unilateral body bend); rather, the shape needs to be sinusoidal (S-shaped). MCoD neurons are a good candidate for implementing this coordinated movement of the trunk in the diagonal dimension, because they are active during slow swimming, and because they are long-distance descending commissural excitatory neurons that make direct connections onto MNs in the caudal region of the contralateral spinal cord^16,17^ (Fig. 1B).

In this study, we tested whether MCoD neurons play a role in the coordinated movements of the trunk in the diagonal dimension, thereby ensuring minimal head yaw displacement during slow swimming. Our laser ablation experiments revealed that MCoD neurons do indeed play the expected role. In the MCoD-ablated larvae, the normal S-shaped body form during swimming was often lost with increased appearance of unilateral C-shaped bends. Concurrently, the head yaw stability was greatly impaired. In addition to swimming, the present study also sheds light on the evolutionarily conserved role of V0v neurons. In mice, long-distance descending commissural V0v neurons have been implicated in interlimb coordination during walking in the diagonal dimension^18^. We suggest that the long-distance descending commissural V0v neurons for the coordinated movements of the body/limbs in the diagonal dimension are the evolutionarily conserved pathway in spinal locomotor circuits.

## Results

### MCoD neurons fire slightly before the nearby ventral root (VR) activity during fictive slow swimming

**Figure 2:**
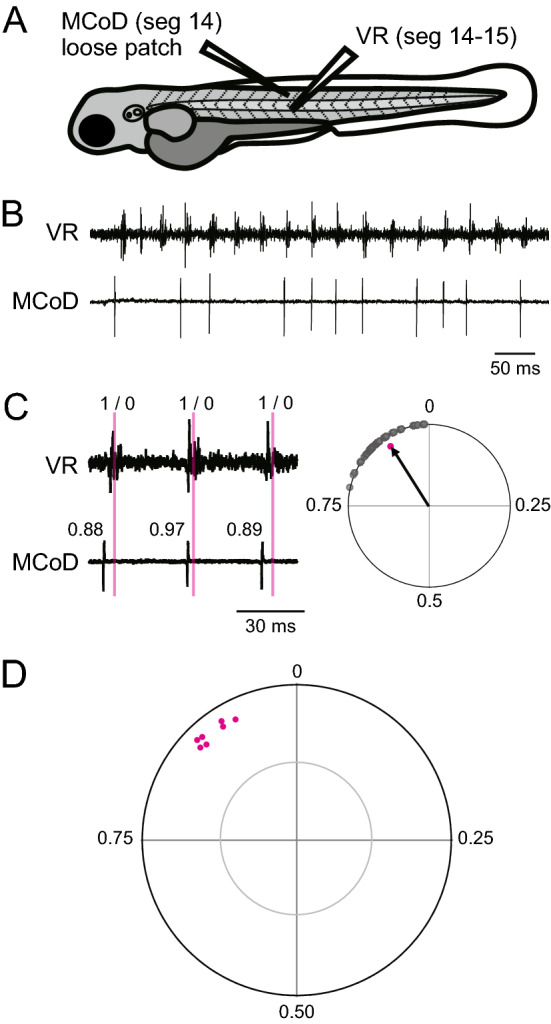
Firing pattern of MCoD neurons during spontaneously occurring fictive slow swimming. (**A**) A schematic illustration of the simultaneous recordings of an MCoD neuron (loose-patch) and ventral root (VR). (**B**) An example of the recording during spontaneously occurring fictive slow swimming. (**C**) A close-up view of two swim cycles. For the phase analysis of spike timings, the middle time point of a VR activity was assigned a phase value of 0, and that of the next VR activity was assigned a phase value of 1. The right panel shows a circular plot of 30 randomly selected spikes relative to VR activity during fictive swimming. The direction of the vector (arrow) shows the mean of the phase value, and the length of the vector shows the strength of the rhythmicity. (**D**) A circular plot showing the spike timing of MCoD neurons (n = 7). The grey circle line marks the 5% significance level.

Previous electrophysiological studies showed that MCoD neurons are rhythmically active during fictive slow swimming, and that the firing timings were generally in phase with nearby motor activity^17,19^. However, a careful phase analysis has not yet been performed. Thus, we addressed this issue by performing loose-patch recordings of MCoD neurons together with motor-nerve (ventral root; VR) recordings. The position of VR recording was set immediately caudal to the MCoD-recording site (Fig. 2A; note that axons of MNs of a given segment exit the spinal cord at the caudal end of the segment). In accordance with previous studies^17,19^, MCoD neurons exhibited rhythmic spiking activities during spontaneously occurring slow swimming (Fig. 2B). For phase analyses of the MCoD spikes, the timing of each spike was represented as its phase in the swim cycle (left panel of Fig. 2C). The phase values of 30 randomly selected spikes are plotted in the circles (grey dots in the right panel of Fig. 2C), and the average value was used as the vector (right panel of Fig. 2C). In this circular plot analysis^20^, the direction of the vector shows the mean of the phase value, whereas the length of the vector shows the strength of the rhythmicity. Figure 2D shows the population data of 7 MCoD neurons (each dot represents the tip of the vector for the neuron examined). The 7 dots show highly clustered distributions, indicating that firing patterns of the MCoD neurons were very similar across the cells. The average phase value was 0.90 ± 0.02. This indicates that the MCoD spikes slightly preceded the nearby VR activities, as is apparent in the representative example shown in Fig. 2C. For all 7 cells, the lengths of the vectors (0.88 ± 0.02) exceeded the significance level (grey circle) with *p* values ranging from 0.0002 to 0.0011, indicating that MCoD neurons fired in a highly rhythmic manner.

During slow swimming, rostro-caudal phase delay is shown to be approximately 0.026 swim cycles per segment^21^. Considering this, the spike timing of MCoD neurons roughly coincides with the peak spike timing of MNs located 4 segments rostral.

### Ablation of MCoD neurons leads to a large increase of head-yaw displacement during spontaneous swimming

**Figure 3:**
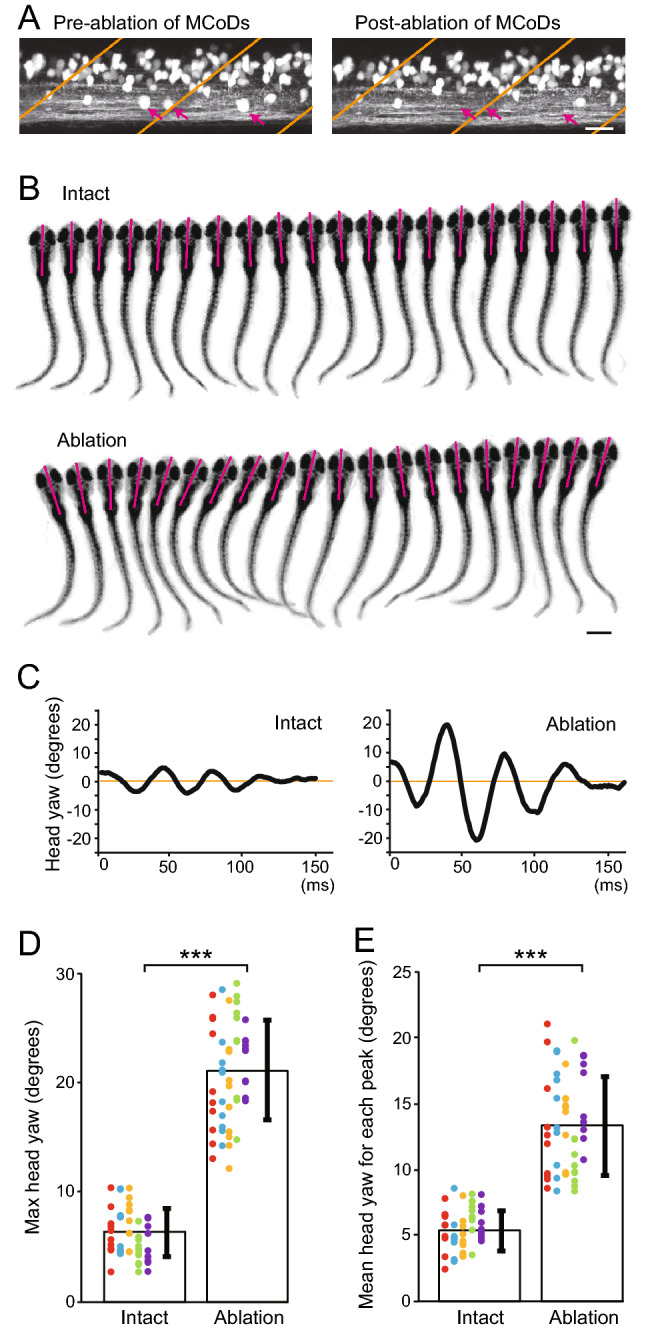
Ablation of MCoD neurons lead to the increase of head-yaw displacement during spontaneous swimming. (**A**) Confocal stacked images of Tg[*evx2*-hs:GFP] fish before (left) and after (right) laser ablation. Images of two hemi-segments are shown. Magenta arrows show MCoD neurons that were chosen for laser ablation. MCoD neurons can be identified by their very ventral location in the spinal cord. Brown lines show boundaries of muscle segments. Scale bar, 20 μm. (**B**) Successive images captured at 1000 frames per second of larval zebrafish swimming. Images of every three frames (3 ms interval) are shown. Magenta bars depict the head directions in each frame. Top, images of an intact fish. Bottom, images of an MCoD-ablated fish. Scale bar, 500 μm. (**C**) Graphs of head yaw angle (y axis) versus time (x-axis) during swimming. Left, intact fish. Right, MCoD-ablated fish. (**D**) Maximum head yaw angle of intact and MCoD-ablated fish during swim bouts. Five fish were examined for each fish type. For each fish, 10 swim bouts were examined. Data obtained from the same fish are color coded. (**E**) Mean head yaw angle for displacement peaks of intact and MCoD-ablated fish during swim bouts. Five fish were examined for each fish type. For each fish, 10 bouts were examined. Data obtained from the same fish are color coded (the same fish as **D**). ***p < 0.001 (two-tailed *t*-test).

To examine the behavioral roles of MCoD neurons in swimming, we performed laser ablation experiments. For each side of the spinal cord at muscle segment 5–17, 15 MCoD neurons (30 MCoD neurons in total) were chosen and ablated using a two-photon microscope (Fig. 3A; note that in Tg[*evx2-hs*:GFP] fish, MCoD neurons can be identified by their ventral and far-lateral location in the spinal cord). Then, we examined the performance of spontaneous swimming using high-speed filming. As control ablation experiments, dorsally located V0v neurons were ablated in a similar manner (Supplementary Fig. 1A). In control ablation animals, spontaneous swimming was virtually indistinguishable from that of wild-type fish (Supplementary movies 1 and 3; Supplementary Fig. 1B–H), indicating that the phenotypes observed in MCoD-ablated fish (described below) were specifically caused by the MCoD ablation, not by the collateral damage of laser ablation.

Figure 3B shows representative examples of successive images during spontaneous swimming of intact and MCoD-ablated fish (see also Supplementary movies 1 and 2). Magenta bars in Fig. 3B depict the head directions in each frame. The discernible difference is the head yaw angle; MCoD-ablated fish exhibited a much larger degree of head yaw displacement. We quantified the head yaw angle during swim bouts. Representative examples are shown in Fig. 3C. In both intact and MCoD-ablated fish, the head yaw angle was larger in the early phase of the swim bout and became smaller in the later phase of the bout. However, there was a large difference in the magnitude: in intact fish, the head yaw angle was around 5 to 6 degrees at maximum whereas in the MCoD-ablated fish, it exceeded 20 degrees (Fig. 3C).

For the quantitative analyses of population data, we measured two parameters: (1) the maximum head yaw angle (absolute value) during a bout, and (2) the mean of the head yaw angles for each peak of displacement (absolute value) during a bout. Figure 3D,E show the data obtained from 5 fish (10 swim bouts were examined from 1 fish). For both parameters, there were large differences. The maximum head yaw angle in the intact fish was 6.40 ± 2.16 degrees whereas that of MCoD-ablated fish was 21.03 ± 4.59 degrees (Fig. 3D); a 3.3-fold increase (*p* = 5.72 × 10^–31^). The mean of the head yaw angles for each peak of displacement in the intact fish was 5.35 ± 1.51 degrees whereas that of MCoD-ablated fish was 13.29 ± 3.69 degrees (Fig. 3E); a 2.5-fold increase (*p* = 2.04 × 10^–21^). In summary, for both (max and mean) parameters, head yaw displacement was greatly increased in MCoD-ablated fish during spontaneous swimming.

**Figure 4:**
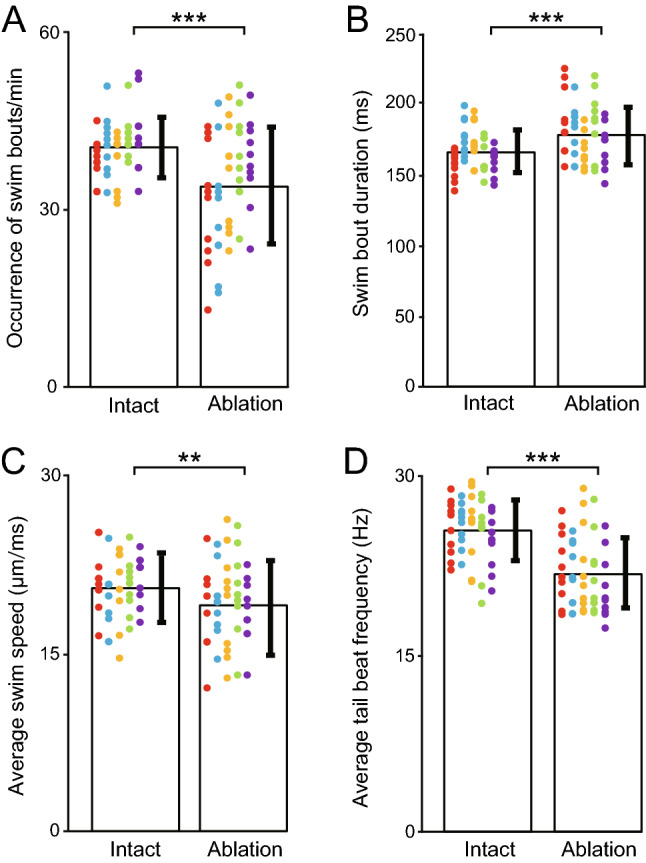
Swim parameters of intact and MCoD-ablated fish. For the analyses of each parameter, five fish were examined for each fish type. For each fish, 10 swim bouts (or a 1-min movie in the case of **A**) were examined. Data obtained from the same fish are color coded (the same fish as Fig. 3D,E). (**A**) Occurrence frequency of swim bouts (per minute) of intact and MCoD-ablated fish. (**B**) Swim bout duration of intact and MCoD-ablated fish. (**C**) Average swim speed of intact and MCoD-ablated fish. (**D**) Average tail beat frequency of intact and MCoD-ablated fish. **p < 0.01, ***p < 0.001 (two-tailed *t*-test).

We also examined several other parameters of swimming. Consistent with a previous study^14^, the occurrence frequency of swim bouts was decreased in MCoD-ablated fish (Fig. 4A; average values: intact fish, 40.50 ± 4.90 times/min; MCoD-ablated fish, 34.76 ± 9.87 times/min; 14.17% decrease; *p* = 0.00018). Swim bout duration was slightly increased (Fig. 4B; average values: intact fish, 164.46 ± 14.78 ms; MCoD-ablated fish, 176.92 ± 20.26 ms; 7.57% increase; *p* = 0.0006). Average swim speed in bouts was slightly decreased (Fig. 4C; average values: intact fish, 21.19 ± 3.34 μm/ms; MCoD-ablated fish, 19.67 ± 4.27 μm/ms; 7.17% decrease; *p* = 0.0083). The average tail beat frequency during swim bouts was slightly decreased (Fig. 4D; average values: intact fish, 25.44 ± 2.48 Hz; MCoD-ablated fish, 21.74 ± 3.08 Hz; 14.54% decrease; *p* = 2.1 × 10^–9^). The number of bends (note that 2 bends constitute 1 swim cycle) during swim bouts was slightly decreased (average values: intact fish, 8.38 ± 1.23 times; MCoD-ablated fish, 7.68 ± 1.39 times; 9.11% decrease; *p* = 0.0057).

### Ablation of MCoD neurons leads to impairment of the S-shaped body bend during swimming

**Figure 5:**
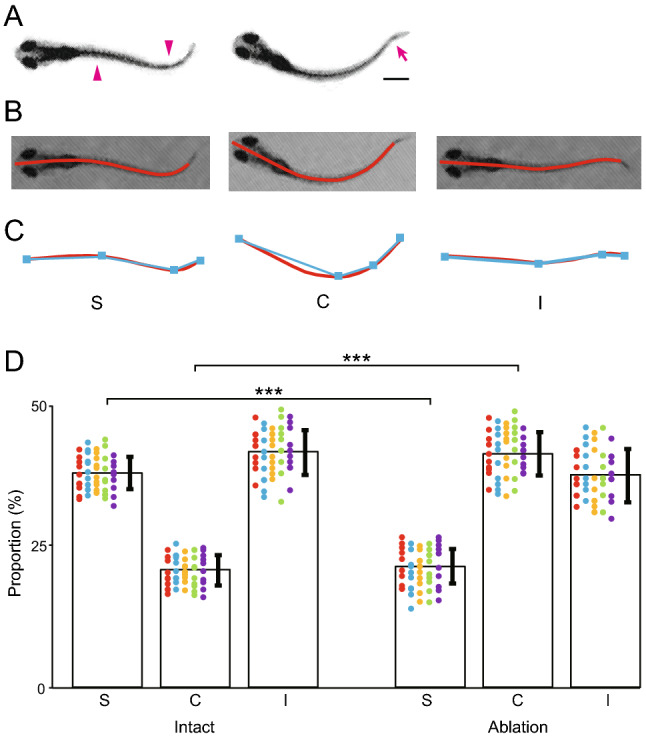
S-shape swim forms are impaired in MCoD-ablated fish. (**A**) Examples of typical swim forms of an intact fish (left) and an MCoD-ablated fish (right). Arrowheads in the left panel show presumed muscle-contraction sites. An arrow in the right panel shows a kinked bend near the tail tip, which is likely produced by passive force from the surrounding water. Scale bar, 500 μm. (**B**) Extractions of the skeletal line representing body shape (red lines). The left panel corresponds to the left panel of (A), and the center panel corresponds to the right panel of (A). The right panel is an image of an intact fish near the end of a swim bout. (**C**) The skeletal line in (C) is fitted to polylines (cyan lines) consisting of four vertices including head and tail termini. (**D**) Histograms of the appearance frequencies of “S”, “C”, and “I” forms in the movie frames of intact fish (left) and MCoD-ablated fish (right). ***p < 0.001 (two-tailed *t*-test).

In the swimming of intact fish, the body shapes in many movie frames appeared to be S-shaped (top panel of Fig. 3B; see also the left panel of Fig. 5A), suggesting that there are two bending sites of the trunk, with each bend to the opposite direction (arrowheads in Fig. 5A). By contrast, in the swimming of MCoD-ablated fish, the body shapes in many movie frames appeared different from those of intact fish; the number of S-shaped body image frames decreased and, concurrently, the number of C-shaped body image frames increased (bottom panel of Fig. 3B; see also the right panel of Fig. 5A). It should be noted that the kinked bending of the far-caudal tail region (arrow in Fig. 5A) is not considered part of the body shape. This is because no trunk muscle exists in this region, and hence, the kinked bending is likely to be produced by passive hydrodynamic force, not by active muscle contraction.

We quantified the occurrence frequency of S-shape and C-shape movie frames with the following method. First, the skeletal line representing body shape was extracted (red lines in Fig. 5B), as was done in our previous paper^22^. Second, the skeletal line was fitted to polylines consisting of four vertices including head and tail termini (cyan lines in Fig. 5C). The relative angles (signed values) of each pair of adjoining edges were then obtained. To judge the body shape of this skeletal model, we multiplied the pair of relative angles and determined whether the resultant value became negative, positive, or very small, which classified the body curve into “S”, “C”, or “I” shapes, respectively (left, center, and right panels, respectively, in Fig. 5C; for further details, see "Methods"). As the amplitude of the body bend tended to be small at the beginning and near the end of swim bouts, movie frames in the first 10 ms and the last 20% of swim bouts were excluded from our analyses.

Figure 5D shows the occurrence frequencies of the “S”, “C”, and “I” shapes in the movie frames of intact and MCoD-ablated fish (for each type, 5 fish were examined; for each fish, 10 swim bouts were examined). Consistent with the observation described above, the proportion of “S” was greatly decreased in MCoD-ablated fish (43.6% decrease; value for intact fish, 37.8 ± 2.9%; value for MCoD-ablated fish, 21.3 ± 3.1%; *p* = 6.9 × 10^–49^); concurrently, the proportion of “C” was greatly increased in MCoD-ablated fish (99.6% increase; value for intact fish, 20.6 ± 2.5%; value for MCoD-ablated fish, 41.2 ± 3.7%; *p* = 2.2 × 10^–54^). The combination of the two tests was performed with Bonferroni correction and statistical significance was supported. The proportion of “I” was slightly decreased in MCoD-ablated fish (9.8% decrease; value for intact fish, 41.6 ± 4.0%; the value for MCoD-ablated fish, 37.5 ± 4.6%; *p* = 8.7 × 10^–6^).

With the large changes in the proportions of “S” and “C” in the MCoD-ablated fish, the “S” versus “C” ratio changed dramatically. In the intact fish, the proportion of “S” is 1.83-fold larger than that of “C.” By contrast, “C” is 1.93-fold larger than that of “S” in the MCoD-ablated fish. The results indicate that MCoD ablation resulted in a frequent loss of the S-shaped swim form, with increased appearances of the C-shaped body form.

### Ablation of MCoD neurons leads to impairment of the tight phase relationship between rostral and caudal motor activities

As a final experiment, we performed electrophysiological recordings of rostral and caudal VRs to show that (1) activities of the rostral VR and caudal VR are anti-phasic (i.e., in-phasic in the diagonal dimension, indicative of S-shape swimming) and (2) this anti-phasic relationship is impaired in MCoD-ablated animals. As noted earlier, rostro-caudal phase delay of VR activities has been shown to be approximately 0.026 swim cycles per segment in the midbody^21^. If this phase delay is maintained along the body axis, VR activities of 19 ~ 20 segment separation would be anti-phasic (around 0.5 phase delay). In light of this, the two recordings sites were set such that they were 19 segments apart.

**Figure 6:**
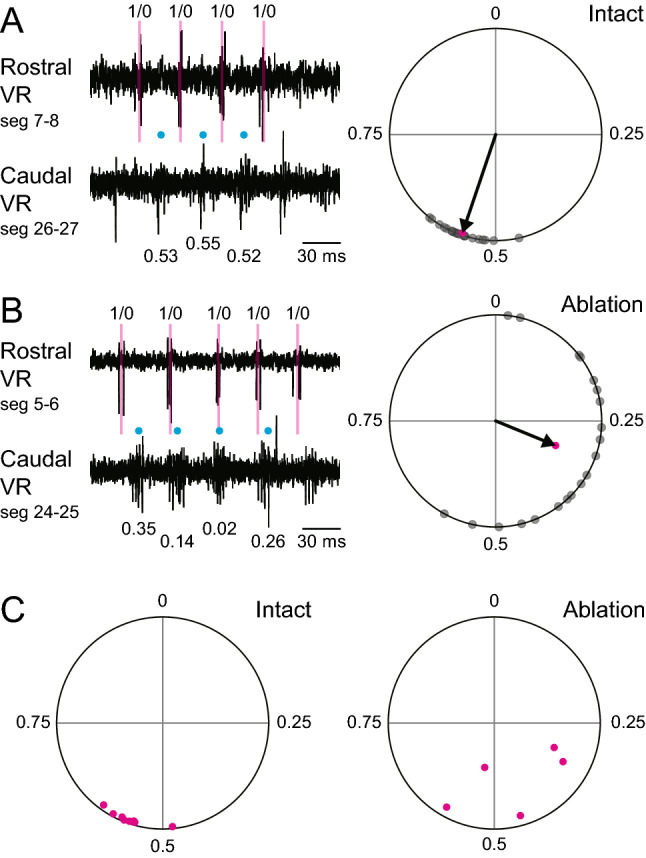
Anti-phasic relationship of the rostral and caudal motor activities is impaired in MCoD-ablated fish. (**A**) Left panel, An example of the dual (rostral and caudal) VR recordings. For the phase analysis, the middle time point of a rostral VR activity was assigned a phase value of 0, and that of the next VR activity was assigned a phase value of 1. Right panel, Circular plot of 20 randomly selected caudal VR activities with respect to rostral VR activity during fictive swimming. (**B**) Same as (A) but for MCoD-ablated fish. (**C**) Left panel, Circular plot for the recordings from intact fish (n = 8). Right panel, Circular plot for the recordings from MCoD-ablated fish (n = 5).

Figure 6A shows a representative example of the recordings, with the left panel being a representative trace and the right panel being the circular plot. As expected, the phase values of the caudal VR activities with respect to the rostral VR activities were close to 0.5. This is also the case in the population data (n = 8) shown in Fig. 6C (left panel). The dots are clustered between 0.48 and 0.60 (0.55 ± 0.03), with all dots located near the circumference of the circle (length of the vectors, 0.97 ± 0.01). These results indicate that the rostral and caudal VR activities were mostly anti-phasic, and that the anti-phase relationship is very tight.

This tight anti-phasic relationship was deteriorated in MCoD-ablated animals, as shown in a representative example in Fig. 6B (left panel). Circular plot analysis of the recordings shows that the dots are more broadly distributed, making the length of the vector shorter (compare the right panels of Fig. 6A,B). The deterioration of the anti-phasic relationship between the rostral and caudal VR activities is apparent in the population data (Fig. 6C). Compared to intact animals (left panel), the phase values obtained in MCoD-ablated animals (n = 5) exhibit broader distributions (0.55 ± 0.03 in intact animals; 0.44 ± 0.11 in MCoD-ablated animals). The lengths of the vectors are shorter (i.e., the dots are located more medially in the circle) in MCoD-ablated animals (0.97 ± 0.01 in intact animals; 0.72 ± 0.21 in MCoD-ablated animals; *p* = 0.052, Welch’s *t*-test). Collectively, the results indicate that MCoD-ablation led to an impairment of the tight anti-phasic relationship between rostral and caudal motor activities.

## Discussion

In this study, we have revealed that MCoD neurons play an important role in allowing fish to perform slow swimming while keeping their heads stable. Before discussing the role of MCoD neurons and the underlying mechanisms in further details, we briefly describe the current understanding of the core neuronal circuits that control swimming.

For vertebrates to execute locomotion, the precise timings and patterns of muscle contractions are generated by the activity of neuron assemblies in the spinal cord that are known as central pattern generators^23–25^ (CPGs). Currently, the widely accepted core components of swimming CPGs in larval aquatic animals such as frog tadpoles^26^ and larval zebrafish are as follows. (1) V2a neurons whose axons mainly descend on the ipsilateral side entrain the activity of MNs and other CPG neurons. The descending axonal trajectory contributes to the caudal-ward wave propagation^27^. (2) V1 neurons whose axons mainly ascend on the ipsilateral side provide recurrent inhibition onto MNs and other CPG neurons, thereby limiting the firing duration of these neurons^28,29^. This recurrent inhibition is also thought to assist the caudal-ward wave propagation^28^. (3) Commissural inhibitory neurons composed of V0d and dI6 neurons provide mid-cycle inhibition onto MNs and other CPG neurons, thereby ensuring the left–right alteration of the motor activity^30^.

In the scheme described above, MCoD neurons (a subclass of V0v neurons) have not been considered a core component of swimming CPG. Indeed, MCoDs have not yet developed in the early developmental stage when embryonic/larval zebrafish acquire the ability to swim. MCoD neurons develop later, and are added to the pre-existing motor circuits^9^. This suggests that the function of MCoD neurons is to provide older larvae with the ability to perform age-appropriate swimming. This led us to hypothesize that MCoD neurons play an important role in enabling fish to swim with their heads kept stable. The present study revealed that this is indeed the case. The laser ablation of MCoD neurons did not deprive larval fish of their ability to perform spontaneous swimming, but it did alter their swimming form such that the stability of the head in the yaw dimension was greatly impaired, with an approximately threefold increase in yaw angle displacement (Fig. 3).

How do the firings of MCoD neurons allow intact larvae to perform slow swimming while keeping their heads stable? The most likely explanation is that the firing activity of MCoD neurons helps create two bending regions in the trunk to form a S-shaped body, such that the net yawing moment force of the trunk that acts to the center of the mass becomes very small (Fig. 1A). The anatomical and physiological properties of MCoD neurons fit this notion. MCoD neurons fire in a highly phasic manner with their spike timing slightly preceding the motor activity located nearby (Fig. 2). Axons of MCoD neurons cross the midline, descend on the contralateral side of the spinal cord, and make monosynaptic excitatory connections onto MNs that are located very caudal (~ 15 segments) to the pre-synaptic MCoD neurons^17^ (Fig. 1B). Assuming that the caudal MNs fire promptly upon receiving excitatory inputs coming from MCoD neurons, participation of MCoD firing activity in the swimming neuronal circuits results in the creation of two bending regions in the diagonal dimension along the body axis, resulting in the S-shaped body form.

In MCoD-ablated larvae, MCoD-mediated crossed-long-distance excitation is lost. Inevitably, motor output patterns need to be generated solely by the core CPG components that consist of ipsilateral descending excitation, ipsilateral ascending inhibition, and crossed inhibition. With this configuration, tight activity coupling in the diagonal dimension is absent. This would result in frequent loss of the S-shaped swim form, with increased appearance of the C-shaped body form. This is exactly what we observed in the swimming of the MCoD-ablated larvae (Fig. 5). With this form of swimming, the net yawing moment force of the trunk that acts to the center of the mass becomes much larger. As a result, the head yaw displacement, which reflects the recoil of the moment force generated by the trunk, was greatly increased (Fig. 3).

Our electrophysiological experiments are in perfect accord with the discussion of the above two paragraphs. Activities of the rostral VR and caudal VR (19 segment apart) were anti-phasic (Fig. 6). Namely, activities of the rostral VR and caudal VR in the diagonal dimension (e.g., rostral left and caudal right) are in-phasic, indicating that fish swim form is indeed S-shaped. In MCoD-ablated animals, the anti-phasic relationship of the rostral and caudal activities was impaired, and the phase relationship became more variable (Fig. 6). The results strongly support the notion that MCoD firing activity plays an important role in generating the S-shaped swim form.

In MCoD-ablated fish, the appearance frequency of the S-shaped swim form was greatly decreased, but not completely abolished (Fig. 5D). There are two possible explanations for this. The first is the possible contribution of residual MCoD neurons. We ablated both sides of 30 MCoD neurons in total at seg 5–17, but it is likely that remaining MCoD neurons were present in the ablated larvae (note that it is not known how many MCoD neurons are present in a larva). These residual MCoD neurons could contribute to the creation of the S-shaped swim form in ablated animals. The second explanation (not mutually exclusive to the first) is that the impairment of the tight coordination of the motor activities in the diagonal dimension made motor activities along the body axis more variable (see Fig. 6), resulting in more random forms of swimming. In this explanation, the S-shaped swim form may appear in a stochastic manner.

What are the advantages of head stability during swimming? One obvious advantage is gaze stabilization, which would help the fish find food and locate dangerous objects during exploration. Another advantage may be that a stable head could reduce drag force during swimming, thereby helping efficient forward propulsion. Consistent with this idea, the average swim speed of the MCoD-ablated fish became slightly slower than that of the intact fish. It should be noted, however, that the general reduction of excitability in the spinal circuits by the ablation of MCoD neurons may also have contributed to the reduction of swim speed (see below).

In addition to head yaw, the ablation of MCoD neurons also affected several parameters that characterize swimming. Consistent with the results of a previous study^17^, the occurrence frequency of swim bouts was decreased (~ 14.2% decrease). This could be explained by the general reduction of excitability in the spinal neuronal circuits due to the ablation of 30 MCoD neurons. The reductions in tail beat frequency (~ 14.5% decrease) and swim speed (~ 7.2% decrease) may also be attributed to the general reduction of excitability. As noted above, the increased drag force that was potentially caused by the increased head yaw displacement may have also contributed to the reduction of swim speed. Unexpectedly, swim bout duration was slightly increased (~ 7.6% increase) in the MCoD-ablated fish, but the reason for this remains unknown. In any case, the magnitudes of the changes in the swim parameters described in Fig. 4 (occurrence frequency of bouts, tail beat frequency, speed, duration of bouts) is smaller than that of the head yaw displacement (an approximately threefold increase). This strongly suggests that the main function of MCoD neurons is to enable fish to keep their head stable during slow swimming.

During faster swimming, head yaw is no longer stable^9,17,31^. In faster swimming, the speed of the rostro-caudal wave propagation is faster than that in slow swimming. In addition, the amplitude of body bend is much larger. Presumably, with this increased speed and bend amplitude, the fish needs to compromise the stability of the head. In this sense, it is reasonable that MCoD neurons are de-recruited in faster swimming^17^; the participation of MCoD neurons in faster swimming would be counterproductive.

As for the developmental maturation of swimming circuits in larval zebrafish, MCoD is not the only class of neurons that are added later in development. It is known that late-born core CPG neurons and MNs are also incorporated into the pre-existing neuronal circuits, and these late-born neurons work together with early-born neurons to produce swimming of various speeds and strengths^7,9^. Here, we have shown that the addition of a new class of neurons (MCoD neurons) enables slow swimming with a new feature: the tight coupling of muscular activities along the body axis in the diagonal dimension. This suggests that addition of new classes of neurons into the pre-existing locomotor circuits during development could play an important role in enabling animals to acquire the abilities to perform new locomotor gaits.

Finally, MCoD neurons belong to a subclass of V0v neurons^30^. Interestingly, in mice, long-distance descending commissural V0v neurons have been implicated in interlimb coordination during walking in the diagonal dimension^18^. Thus, the roles of these neurons during locomotion are similar to those of MCoD neurons. Taken together, the involvement of long-distance descending V0v neurons for the coordinated movements of the body/limbs in the diagonal dimension is suggested to be the evolutionarily conserved pathway in spinal locomotor circuits. Evolutionarily, swimming movement appeared earlier than walking movement. It is thus tempting to speculate that the MCoD-like neurons that were already present in a common ancestor of vertebrates served as a foundation for evolving long-distance descending commissural V0v neurons that play roles in interlimb coordination during walking.

## Methods

### Fish care and strains

Zebrafish adults, embryos, and larvae were maintained at 28.5 °C. All protocols for this study were approved by the animal care and use committees of the National Institutes of Natural Sciences. The experiments were performed at the Center for Animal Resources and Collaborative Study in Okazaki, in accordance with relevant guidelines regulations. This study was carried out in compliance with the ARRIVE guidelines for involvement of animals (fish). Animals were staged according to days post fertilization (dpf). Wild-type or Tg[*evx2-hs*:GFP] fish were used in this study. The latter was generated in this study using the CRISPR/Cas9-mediated knock-in technique^32^. The donor plasmid used was Mbait-hsp70:GFP^27^. The sgRNA sequence for targeting the *evx2* locus was the same as the one described in Kimura et al. (2014)^32^.

### Electrophysiology

In vivo loose-patch and ventral root (VR) recordings were performed as described previously^22,29,30^. Larvae of Tg[*evx2-hs*:GFP] (heterozygous) at 5 dpf were immobilized by soaking in the neuromuscular blocker d-tubocurarine (0.1 mg/ml in distilled water) for 5 to 15 min, then pinned through the notochord to a Sylgard-coated, glass-bottomed dish with short pieces of fine tungsten pins. Animals were then covered with extracellular recording solution that contained (in mM) 134 NaCl, 2.9 KCl, 1.2 MgCl_2_, 2.1 CaCl_2_, 10 HEPES, 0.01 d-tubocurarine, and 10 glucose, adjusted to pH 7.8 with NaOH. The skin covering mid-body was removed with a pair of forceps. Then, muscle fibers at muscle segment 14 were carefully removed manually with a tungsten needle. For all electrophysiology experiments, the preparations were observed using a water immersion objective (40x; NA, 0.80; Olympus) on an upright microscope (BX51WI; Olympus) fitted with differential interference contrast (DIC) optics. MCoD neurons located at the dissected muscle segment were targeted for loose-patch recordings. We analyzed fictive swimming that occurred spontaneously. VR recordings of axial motor nerves were made immediately caudal to the muscle segment of the MCoD-recording site (between muscle segment 14 and 15). For the rostral and caudal VR recordings, two recording sites were set 19 segments apart (the rostral recording sites were segment 6 to 9). Electrodes for VR recordings (tip diameter, 30–50 μm) and loose-patch recordings (resistance, 9–12 MΩ) were filled with extracellular recording solution. Electrophysiological recordings were performed using MultiClamp700B amplifiers and a Digidata1440A digitizer (Molecular Devices) with a sampling rate of 20 kHz. VR signal was filtered with a low- and high-frequency cutoff at 100 and 4000 Hz, respectively. Loose-patch signal was filtered with a high-frequency cutoff at 4000 Hz.

### Circular plot analysis

Electrophysiological data were analyzed with DataView (software by William Heitler, University of St. Andrews) and Excel (Microsoft). VR recordings were rectified and smoothened. To detect each instance of VR activity, a threshold value was set by visual inspection. For the phase analysis, the middle time point of a VR activity was assigned a phase value of 0, and that of the next VR activity was assigned a phase value of 1. In determining the frequency of swimming, the interval between time points 0 and 1 was defined as the cycle period. Tail beat frequency was the inverse of the cycle period.

Circular plot analysis for the firings of MCoD neurons was performed essentially as described previously^20^ to provide a statistical measure of the coupling between neuronal firing and the phase of VR bursts. Spikes that occurred during smooth slow swimming (24–30 Hz) were subject to analysis. For each of the recorded cells, 30 spikes that fulfilled the criterion described above were randomly selected by a computer, and the phase values of these selected spikes were determined and plotted in a circle.

Circular plot analysis for the caudal VR activities with respect to the rostral VR activities was performed in a similar manner. VR activities during smooth slow swimming in which swimming frequency was below 32 Hz were subject to analysis. We noticed that the intensity of fictive swimming was variable among swim episodes: in some episodes the intensity was high (large duty cycles and large amplitudes of VR signals) while in other episodes the intensity was low (smaller duty cycles and smaller amplitudes of VR signals). In a natural condition (not in a fictive preparation in which the fish is lying on its side), spontaneous swimming represents the weakest swimming. This suggests that low-intensity swimming in a fictive preparation paralleled spontaneous swimming in a natural condition. In this context, we analyzed VR activities that occurred during low-intensity swim episodes. The judgment of swimming intensity was done through visual inspection of the rostral VR activities. Twenty VR activities were randomly selected by a computer, and the phase values of the caudal ones with respect to the rostral were determined and plotted in a circle.

### Laser ablation

Laser ablation of MCoD neurons was performed in 4 dpf larvae of the Tg[*evx2-hs*:GFP] line. Larvae were anesthetized and laterally embedded in 1.5% low melting-point agarose (Thermo Fisher Scientific). Then, the sample was placed under a multiphoton inverted microscope (Leica TCS SP8 MP). MCoD neurons located at segment 5–17 were subjected to laser ablation. MCoD neurons were identified by their conspicuous location among GFP-positive V0v neurons: they are located at the ventral and far-lateral region of the spinal cord. Within the target region (segment 5–17), 15 MCoD neurons (1–2 cells per segment) were unilaterally (the side near the objective lens) photo-ablated using a two-photon laser (wavelength 900 nm; InSight DeepSee, Spectra Physics) and a 40 × objective. The laser was focused on each MCoD neuron with 48 × zoom. A laser scan with a duration between 87 and 290 ms was run 1 or 2 times. Only when fluorescence remained after the first scan was the second scan run. Laser power was 52 mW at the objective lens output. After the unilateral ablation, the sample was re-mounted in agarose after flipping to the opposite side. Then, 15 MCoD neurons on the opposite side were laser-ablated in a similar manner. After the bilateral ablation, larvae were allowed to recover until 5 dpf, and were then used for behavioral experiments. Successful ablations were verified after the behavioral experiments by checking for the absence of GFP fluorescence. For control ablation experiments, the same number of GFP-positive neurons (15 cells per hemi-segment) located in the dorsal column of V0v neurons at segment 5–17 were ablated as described above.

### Behavioral analyses

Behavioral experiments were performed essentially as described previously^22,34,35^ using 5 dpf larvae. Sequential images of swimming were captured at 1000 frames/s with a high-speed camera (FASTCAM-ultima1024; Photron). Fish were filmed from their dorsal side. We analyzed swimming that occurred spontaneously.

The extraction of the body shape model (skeletal line) of each fish was performed as described in Uemura et al. (2020)^22^. Briefly, a small sample of points in the eye region that were visible at the lowest pixel intensity were identified in image frames and used to define a scanning line that was orthogonal to the line between the eyes. At intervals of eight pixels along the line between the eyes, pixel intensities within the arc-shaped region were sampled and the borders of left/right sides were determined by finding the change points for the variance in their intensities. This procedure was repeatedly applied with shifted sampling arc positions to reduce the proportion of erroneous body-border detections. Segments of cubic spline functions were then obtained by applying a smoothing spline interpolation method to the center at the pairs of border positions (i.e., along the midline). The output is treated as the skeletal line.

The parameters for swimming were determined in the following manner.

*The start and end of a swim bout*: The start of a swim bout was defined by the initiation of body movement. The end of a swim bout was defined by the discontinuation of body movement. Due to the low Reynolds number of spontaneous swimming in larval zebrafish^36^, there was virtually no inertial forward advancement of the body after the termination of body movement. The movie frame that corresponds to immediately before the onset of movement was defined as time 0.

*Direction of a swim bout*: As a reference position to represent fish, we used the midpoint of the left and right eyes. The direction of a swim bout was determined by drawing a straight line from the starting point to the end point.

*Head yaw angle*: The anterior part of the body shape model (described above), which is approximately 30% of the total body length, was subjected to principal component analysis (PCA), and a vector of the first principal component was determined as the head direction. The head yaw angle was determined by taking an angle of the head direction against a fitted line to the trajectory for the medial points of the eyes throughout the movie frames.

*Swim bout duration*: The duration of a swim bout was defined by the lapsed time from time 0 (the movie frame that corresponds to immediately before the onset of movement) to the end of movement.

*Average tail beat frequency*: The average tail beat frequency in a swim bout was calculated in the following manner. First, the number of swim cycles in a bout were determined by the visual inspection of the movie. Then, the average cycle period of swimming was calculated by dividing the swim bout duration with the number of swim cycles. The tail beat frequency is the inverse of the cycle period.

*Average swim speed*: The average swim speed of a bout was determined by dividing the travel distance with the duration of the swim bout.

*Number of swim bout*: The number of swim bouts was determined by a visual inspection of high-speed movies at 2 × speed. For each fish, a 10-min corresponding movie was inspected, and it was binned into ten (1 min for each bin).

The body bending property of the fish was classified as either “S”, “I”, or “C” based on how similar the body shaped looked to each of the character contours. First, a skeletal line was fitted to polylines consisting of four vertices, including the head and tail termini, and then the relative angles (signed values) of each pair of adjoining edges were obtained. Then, a pair of relative angles (radian) at the medial two vertices of the polyline was multiplied. The classification was performed as to whether the product is negative, positive, or very small (absolute value of the product was less than 0.1), which sorted the body curve into “S”, “C”, or “I”, respectively.

### Statistics

Results are presented as the mean ± standard deviation (SD). Statistical significance was assessed using the Student’s *t*-test unless otherwise noted.

## Supplementary Information

Supplementary Information 1.

Supplementary Information 2.

Supplementary Video 1.

Supplementary Video 2.

Supplementary Video 3.
