# Supplementary material for: Long descending commissural V0v neurons ensure coordinated swimming movements along the body axis in larval zebrafish
Source: Sci Rep. 2022 Mar 14;12:4348. doi: 10.1038/s41598-022-08283-0 (PMC8921517; doi:10.1038/s41598-022-08283-0)
Supplement: Supplementary file 1 — Supplementary Information 1. [file 41598_2022_8283_MOESM1_ESM.pdf]

Legends to Supplementary Movies

Supplementary Movie 1

Spontaneous swim bout of an intact fish

Supplementary Movie 2

Spontaneous swim bout of an MCoD-ablated fish

Supplementary Movie 3

Spontaneous swim bout of a dorsal-V0v-ablated fish
