# Supplementary material for: Long descending commissural V0v neurons ensure coordinated swimming movements along the body axis in larval zebrafish
Source: Sci Rep. 2022 Mar 14;12:4348. doi: 10.1038/s41598-022-08283-0 (PMC8921517; doi:10.1038/s41598-022-08283-0)
Supplement: Supplementary file 2 — Supplementary Information 2. [file 41598_2022_8283_MOESM2_ESM.pdf]

Supplementary information

Long descending commissural V0v neurons ensure coordinated swimming movements along the body axis in larval zebrafish

Kohei Kawano[1, 2], Kagayaki Kato[1, 2], Takumi Sugioka[1, 2], Yukiko Kimura[1, 2], Masashi Tanimoto[1, 2], and Shin-ichi Higashijima[1, 2*]

[1]National Institutes of Natural Sciences, Exploratory Research Center on Life and Living Systems (ExCELLS), National Institute for Basic Biology, Okazaki, Aichi 444-8787, Japan
[2]Graduate University for Advanced Studies (SOKENDAI), Okazaki, Aichi 444-8787, Japan

*Correspondence: shigashi@nibb.ac.jp

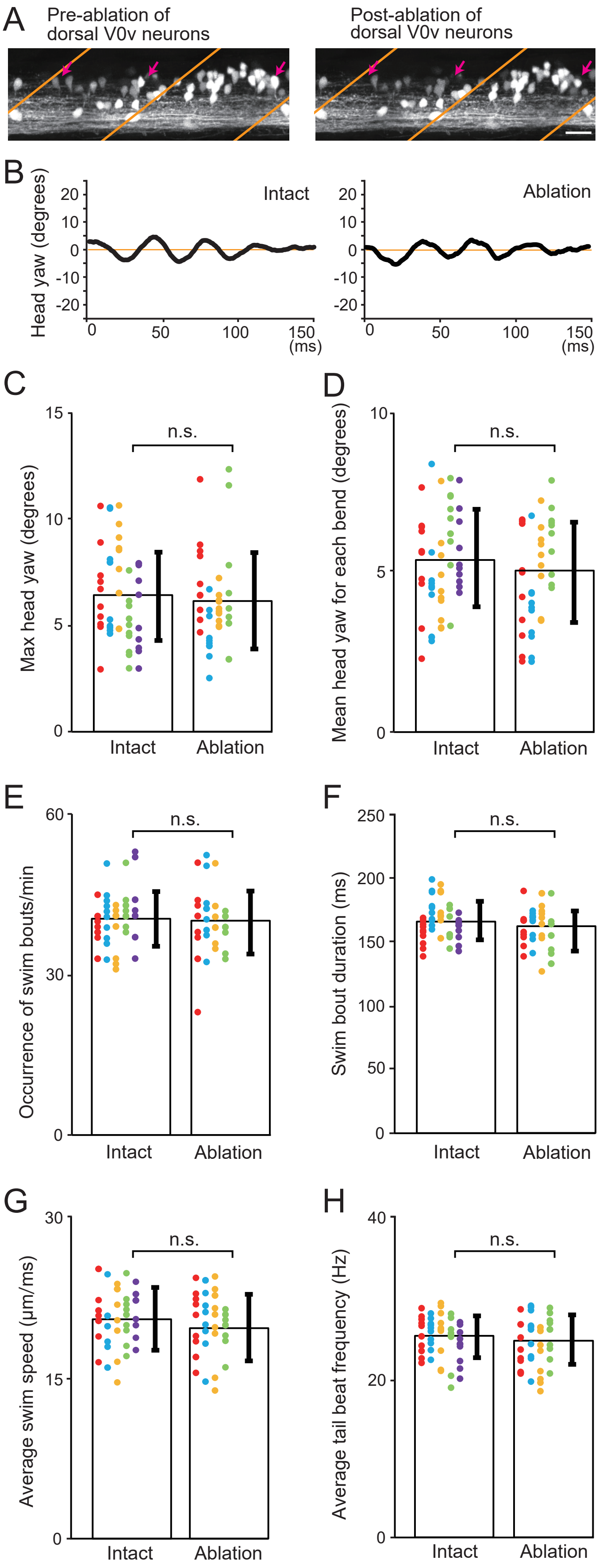

A
Pre-ablation of dorsal V0v neurons
Post-ablation of dorsal V0v neurons
B
Head yaw (degrees)
Intact
Ablation
(ms)
C
Max head yaw (degrees)
n.s.
Intact
Ablation
D
Mean head yaw for each bend (degrees)
n.s.
Intact
Ablation
E
Occurrence of swim bouts/min
n.s.
Intact
Ablation
F
Swim bout duration (ms)
n.s.
Intact
Ablation
G
Average swim speed (μm/ms)
n.s.
Intact
Ablation
H
Average tail beat frequency (Hz)
n.s.
Intact
Ablation

Supplementary Figure 1

Spontaneous swimming in dorsal-V0v-ablated fish did not show any obvious defects

(**A**) Laser ablation of dorsally located V0v neurons. Confocal stacked images of Tg[*evx2*-hs:GFP] fish before (left) and after (right) laser ablation. Images of two hemi-segments are shown. Magenta arrows show dorsally located V0v neurons that were chosen for laser ablation. Brown lines show boundaries of muscle segments. Scale bar, 20 μm. (**B**) Graphs of head yaw angle (y axis) versus time (x-axis) during swimming. Left, intact fish. Right, dorsal-V0v-ablated fish. For the rest of the panels (C-H), five fish were examined for each fish type. For each fish, 10 swim bouts (or a 1-min movie in the case of E) were examined. Data obtained from the same fish are color coded. (**C**) Maximum head yaw angle of intact and dorsal-ablated fish during swim bouts. Average values: intact fish, 6.40 ± 2.16; dorsal-V0v-ablated fish, 6.12 ± 2.18. Statistically not significant (n.s.; $p = 0.54$). (**D**) Mean head yaw angle for displacement peaks of intact and dorsal-V0v-ablated fish during swim bouts. Average values: intact fish, 5.35 ± 1.51 degrees; dorsal-V0v-ablated fish, 4.98 ± 1.56 degrees. Statistically not significant (n.s.; $p = 0.18$). (**E**) Occurrence frequency of swim bouts (per min) of intact and dorsal-V0v-ablated fish. Average values: intact fish, 40.50 ± 4.90 times/min; dorsal-V0v-ablated fish, 40.15 ± 5.59 times/min. Statistically not significant (n.s.; $p = 0.75$). (**F**) Swim bout duration of intact and dorsal-V0v-ablated fish. Average values: intact fish, 40.50 ± 4.90 ms; dorsal-V0v-ablated fish, 40.15 ± 5.59 ms. Statistically not significant (n.s.; $p = 0.75$). (**G**) Average swim speed in bouts of intact and dorsal-V0v-ablated fish. Average values: intact fish, 146.46 ± 14.78 μm/ms; dorsal-V0v-ablated fish, 161.15 ± 16.07 μm/ms. Statistically not significant (n.s.; $p = 0.31$). (**H**) Average tail beat frequency in bouts of intact and dorsal-V0v-ablated fish. Average values: intact fish, 25.44 ± 2.48 Hz; dorsal-V0v-ablated fish, 24.76 ± 3.05 Hz. Statistically not significant (n.s.; $p = 0.21$).
